# Social–emotional learning program: a community-based case-controlled study

**DOI:** 10.20935/mhealthwellb7308

**Published:** 2024-08-13

**Authors:** Kit Knier, Taylor Harrison, Michelle Grady, Andrew Brock, Gauri Sood, Debbie Fuehrer, Amit Sood, Chris Pierret

**Affiliations:** 1 Mayo Clinic Graduate School of Biomedical Sciences, Mayo Clinic, Rochester, MN 55905, USA.; 2 Mayo Clinic Medical Scientist Training Program, Mayo Clinic, Rochester, MN 55905, USA.; 3 Bioinformatics and Computational Biology Program, University of Minnesota, Minneapolis, MN 55455, USA.; 4 Department of Physiology and Biomedical Engineering, Mayo Clinic, Rochester, MN 55905, USA.; 5 Independent Researcher, Eyota, MN 55934, USA.; 6 Global Center for Resiliency and Wellbeing, Rochester, MN 55905, USA.; 7 Mayo Clinic College of Medicine and Science, Mayo Clinic, Rochester, MN 55905, USA.; 8 Department of Biochemistry and Molecular Biology, Mayo Clinic, Rochester, MN 55905, USA.

**Keywords:** child well-being, mindfulness, child behavior, emotions, education, program evaluation

## Abstract

HappiGenius is a mindfulness-based social–emotional learning (SEL) program developed and initially deployed in the context of COVID-19. Novel features of the HappiGenius program include the use of behavioral practice and activities to enhance traditional classroom-based learning, the capability to teach in a remote setting, and a focus on internalizing behaviors in addition to traditionally targeted externalizing behaviors. A pilot study of four third-grade classrooms completed in the previous academic year showed promising results for improvements in students’ experience of positive affect, self-compassion, total difficulties, and prosocial behaviors. This quasi-experimental case-control study was conducted in 14 third-grade classrooms across four schools in a midsize Midwestern US city. We hypothesized that HappiGenius would lead to improvements in positive affect, self-compassion, and mindfulness, in addition to other social behaviors measured by quantitative standardized scales. We used qualitative methodology to determine whether HappiGenius was feasible and acceptable to teachers for use in the classroom. While there was no statistically significant change in positive affect, self-compassion, or mindfulness, there was meaningful improvement in student internalizing problems and peer problems. Analysis of qualitative data showed the importance of timing during the year to maximize classroom benefits like establishing behavioral expectations early and reducing problem behaviors. The study represents a step in the direction of evaluating the implementation and outcomes of a new social–emotional learning program developed with deliberate attention to internalizing problems alongside traditionally studied externalizing problems.

## 1. Introduction

Children’s and adolescents’ mental, emotional, and behavioral health has been on the decline in the last five to six decades [1, 2]. Perhaps some of the most startling emerging data is the increasing rate of suicidal ideation and completed suicides among young people. From 2011 to 2020, children aged five to nine experienced a 32.9% annual increase in suicide-related hospital visits (0.1–3.5 per 1,000 capita), with even greater overall increases in the 10–14 years age group [3]. From 2005 to 2020, the Centers for Disease Control and Prevention (CDC) reported a 2.4-fold increase in deaths by suicide in the under-15 age group, and in the 2018 to 2022 report, death by suicide is second only to accidental trauma among 10- to 14-year-olds [1]. The COVID-19 pandemic led to additional stress, worry, and feelings of helplessness in young people [1, 4]. Lifestyle disruptions, including school closures, physical distancing, quarantine, and isolation, have been associated with rising depressive and anxious symptoms among children [4–8].

During this time, health promotion and prevention, rather than disease identification and treatment, have increasingly been recognized as essential to improving the health of children in our communities [1, 9]. Ongoing programs directed at parents and teachers, or delivered in educational settings, seem to be more effective than interventions delivered to children during clinic visits [10, 11]. In 2019, the National Academies of Sciences, Engineering, and Medicine released a consensus report on children’s mental, emotional, and behavioral developments [12]. The report outlines child development, risks, and evidence-based opportunities across an individual’s life course. The life course approach acknowledges the cumulative nature of effects beginning even before birth and recognizes that multiple levels of the social–ecological model of health—beyond the individual, including home, school, neighborhood, community, and policy-making bodies—impact development [9, 12]. This approach is one way to understand and begin to address well-being. Well-being is a subjective state of mind arising from an individual’s thoughts and actions, including an expectation of positive outcomes and the ability to adapt to one’s circumstances [13–15]. Well-being is a concept distinct from but important to the development of health and wellness; evidence suggests that cultivating well-being throughout the life course may promote mental, emotional, and behavioral health in the long term [14–16].

Social–emotional learning (SEL) programs are specialized curricula designed to develop and support students’ well-being. In the mid-1990s, the Collaborative for Academic, Social, and Emotional Learning (CASEL) was formed by cross-disciplinary leaders to establish guidelines for SEL programming. The CASEL standards have been iteratively revised based on the available research evidence to include the following: (i) the program must have a positive behavioral outcome, (ii) improved academic performance, (iii) improved positive social behavior, (iv) reduced problem behaviors, and (v) reduced emotional distress [17]. Research on the long-term outcomes of SEL programs delivered to elementary-age children has identified broad benefits for individual well-being, including improved attendance, higher high school graduation rates, safer sex practices, better peer relationships, and decreased drug use, arrests, and juvenile justice involvement. Additionally, there is less frequent diagnosis of conduct disorders and other clinical disorders, such as substance abuse, persisting for more than nine years after program delivery [18–21]. SEL programs have primarily been employed in the school setting, though research shows that reinforcing these concepts in the home environment with caregivers and outside the classroom through cultural shifts within the school strengthen beneficial outcomes [12, 22]. SEL programs have traditionally addressed behavioral and emotional health, though the most recent guideline update from CASEL outlines goals for addressing internalizing tendencies and improving equity and cultural relevance for SEL programs [17].

Mindfulness strategies are a relatively new addition to SEL programming that may address internalizing tendencies. The exact mechanism by which mindfulness works is not known, but one hypothesis is that mindfulness practices improve distress tolerance and emotional regulation, allowing the user to moderate their internal experience and attain goals despite times of stress [12, 23, 24]. Many of the attributes targeted by mindfulness practice may contribute to developing CASEL’s target competencies and addressing internalizing tendencies. For example, understanding one’s own mind and awareness of current emotions promote self-awareness. Additionally, mindfulness practices disrupt automatic responses, reduce reactivity to stressful situations, and develop the inhibitory self-control necessary for self-management and responsible decision-making. Repeatedly practicing reorientation to a specific focus (e.g., the breath) can strengthen attention, which is important to self-management and relationship skills, and together, awareness and control over emotional responses to the environment may increase resilience and decrease negative affect, rumination, and symptoms of anxious or depressive thinking [12, 25]. Studies of mindfulness-based SEL programming in the USA and North America have mainly focused on pre-K, kindergarten, and fourth- to fifth-grade students. Meta-analyses of mindfulness programs delivered in the school setting demonstrate consistent improvements in executive function and attention and reductions in depression, anxiety, stress, and negative behaviors in children and teens [21, 26].

HappiGenius is an affordable and scalable mindfulness-based SEL program that was developed by a cross-disciplinary team of educators, clinicians, and psychologists. The HappiGenius program has the potential to influence internalizing problems and behavior through the inclusion of mindfulness and resiliency training. Each of the 12 lessons includes the four core skills: gratitude, kindness, focus, and calming down (full descriptions provided in Table S1, Supplementary material, of the 2023 pilot study) [27]. In addition to these four skills, another aspect that sets HappiGenius apart from some of the other available programs is its delivery method. Inconsistent reporting of implementation and delivery of programming has been a concern in the current research base for mindfulness programs in schools [12]. The HappiGenius lessons are delivered by trainers who have undergone a dedicated two-hour-long workshop on the program’s rationale, design, and delivery. The core components of the lessons include interactive videos, supplemented by discussions, coloring activities, and option weekly at-home exercises. This approach ensures that all students receive the same core components, regardless of variations between classes and teachers.

The HappiGenius program’s targets align with CASEL standards, including the behavioral outcome of building student attention, improving academic performance by enhancing learning, improving social skills, reducing conduct problems, and reducing emotional problems through access to uplifting emotions, mindfulness, and self-kindness. In the literature, outcome measures used to assess SEL programs included cognitive (executive function, attention, school self-concept, math achievement, and literacy), social–emotional skills (self-management, emotional control, social awareness, empathy and perspective taking, kindness, gratitude, peer and group relationships, and classroom climate), symptoms (internalizing issues, e.g., depression and anxiety, and externalizing issues, e.g., aggression, conduct, and hyperactivity), and mindfulness [12, 28]. Due to the restrictions of this research setting and the limited attentional abilities of younger children, we opted to focus our study on the components we thought were most foundational: social–emotional skills, internalizing and externalizing tendencies, and mindfulness. A pilot study of the HappiGenius program showed significant improvements in positive emotions, self-compassion, total difficulties, emotional problems, peer problems, conduct problems, and prosociality [27].

To further study the effectiveness of HappiGenius, we conducted the prospective quasi-experimental case-control study presented here. We sought to examine whether HappiGenius improves the competencies first studied in the pilot cohort from baseline to the end of the program using traditional quantitative survey methodology, comparing outcomes with a control group receiving class as usual. We opted for an effectiveness design to better reflect the real-world variability and evaluate any impact of the program, versus an efficacy design which provides control for variability and produces larger effect sizes but does not always translate outside of a tightly controlled research environment. Beyond applying the quantitative measures used in the pilot study to a larger group of students across multiple schools, we also pursued the question of the HappiGenius program’s implementation feasibility and acceptability to teachers and their students for use in the classroom using qualitative analysis of semi-structured teacher interviews.

## 2. Materials and methods

### 2.1. Study design

This quasi-experimental case-control study included a prospective quantitative survey-based component and a qualitative interview-based study. Across one public school district in a midsize Midwestern US city, 14 third-grade classrooms from four schools participated in the study. The study, which included student surveys, teacher surveys, and teacher interviews, was approved by the Institutional Review Board (IRB) and complied with all human subject protection regulations. Prior to the start of the survey study, written parental consent was obtained. Student assent was also sought prior to each survey. Students whose parents or legal guardian(s) did not consent to the study or students who did not assent were excluded from the data analysis.

### 2.2. Study site and participants

#### 2.2.1. Setting

In the week before the start of the program, a member of the research team met with each classroom for a dedicated 30-minute time block to describe the research, discuss potential risks and benefits, and review the concept of assent. The preprogram survey was administered one to five school days before the start of the program to both case and control classrooms. The 12 lessons of 45 minutes each were delivered in sequence once a week over the course of the semester. The post-program survey was delivered within five school days of the final lesson. The three-month survey was delivered three months following the post-program survey. Teacher participation in the interviews was solicited by email using an IRB-approved script two weeks in advance. Teachers who agreed to participate were interviewed by K.K. in their classrooms before or after the school day. Interviews lasted approximately 20 minutes each and were audio recorded with the permission of each teacher.

#### 2.2.2. Delivery agents

Teachers participated in a two-hour training session and quiz to become certified as HappiGenius instructors through the Global Center for Resiliency and Wellbeing [29]. The classroom teachers taught all 12 HappiGenius lessons to their students within the school day.

#### 2.2.3. Participants

Student participants were from 14 third-grade classrooms across four public elementary schools in a midsize Midwestern US city. Available demographics are outlined in Table S1, Supplementary material. The inclusion criterion was student enrollment in a third-grade classroom receiving HappiGenius. Exclusion criteria included having no written informed consent from a parent or guardian and students who did not assent to the surveys. Within the 14 participating classrooms, 131 students had written parental consent and provided student assent. One student transferred schools before conclusion of the program, leaving a total of 130 student participants.

Of the teachers from the 14 participating classrooms, seven agreed to provide feedback at the end of the academic year. Of those, four participated in semi-structured interviews in person and agreed to record the session; one participated in an in-person semi-structured interview but declined recording the session, so field notes were taken; and two volunteered to provide feedback by email. All four of the participating schools were represented by at least one and up to three teachers.

### 2.3. Data collection and analysis

Variables were chosen from the HappiGenius program targets aligned with CASEL standards, as described in the pilot study ([27], Table 1). Briefly, the survey instruments included validated measures of positive affect [Positive and Negative Affect Scale for Children Positive Affect only (PANAS-C-PA); *α* = 0.84–0.87] [30–32], self-compassion [Self-Compassion Scale for Children (SCS-C); *α* = 0.57–0.63] [33], mindful self-awareness [Mindful Attention Awareness Scale for Children (MAAS-C); *α* = 0.89–0.90] [25], and student attention and social skills [teacher-completed Strengths and Difficulties Questionnaire (SDQ-T); *α* = 0.88–0.92] [34, 35]. Complete details of the measures and validation are included in Section Ia, Supplementary materials along with post hoc correlation analyses performed on selected baseline measures (Table S2 and Figure S1a–e, Supplementary material). Students with incomplete responses to either the pre- or post-program self-report surveys were excluded from the analysis. Quantitative analysis was conducted in a blinded manner. Before analysis, the data were blinded by removing individual and group identifiers. Descriptive statistical reports were generated for each survey instrument. For data that met assumptions of normality, outcomes are reported as means (standard deviation), and multivariable linear regression modeling was used to determine whether HappiGenius or the class-as-usual (CAU) conditions influenced survey outcomes. Non-normally distributed outcomes are reported as medians (interquartile range) rather than means. These outcomes were evaluated using Wilcoxon matched-pairs signed-rank tests, except where indicated otherwise. A type 1 error rate of 5% was used to assess statistical significance.

For semi-structured interviews with teachers at conclusion of the school year, core questions to assess the feasibility and acceptability of the program for teachers and their students from the teachers’ perspective were determined in advance and used to guide the conversation (questions provided in Section Ib, Supplementary materials). T.H. converted the content of the recorded interviews into typewritten text, and K.K. typed the content of field notes, which were uploaded along with the emailed feedback to a common qualitative analysis software program for organization and analysis. Thematic analysis was used to identify core concepts and understand the qualitative data. Initially, the text was reviewed to extract high-level core concepts. Reviewers K.K. and T.H. identified core concepts independently and then cross-referenced the concepts to identify agreements and resolve disagreements. The core concepts were recorded in a code book and used to organize distinct comments. Comments associated with each core concept were iteratively reviewed and analyzed to identify sub-concepts and patterns present within the concept. The data were reviewed again to identify patterns and relationships between sub-concepts, first independently and then through crossreferencing between K.K. and T.H. to again identify agreements and resolve any disagreements. A diagram was generated to represent the results, including the core concepts and the relationships between sub-concepts, which sometimes straddled multiple core concepts. Representative quotes were identified and collated in a summary of results.

## 3. Results

### 3.1. Quantitative results

The student- and teacher-reported survey measures were similar between HappiGenius classrooms and CAU at baseline (Table S3, Supplementary material).

**Figure 1:**
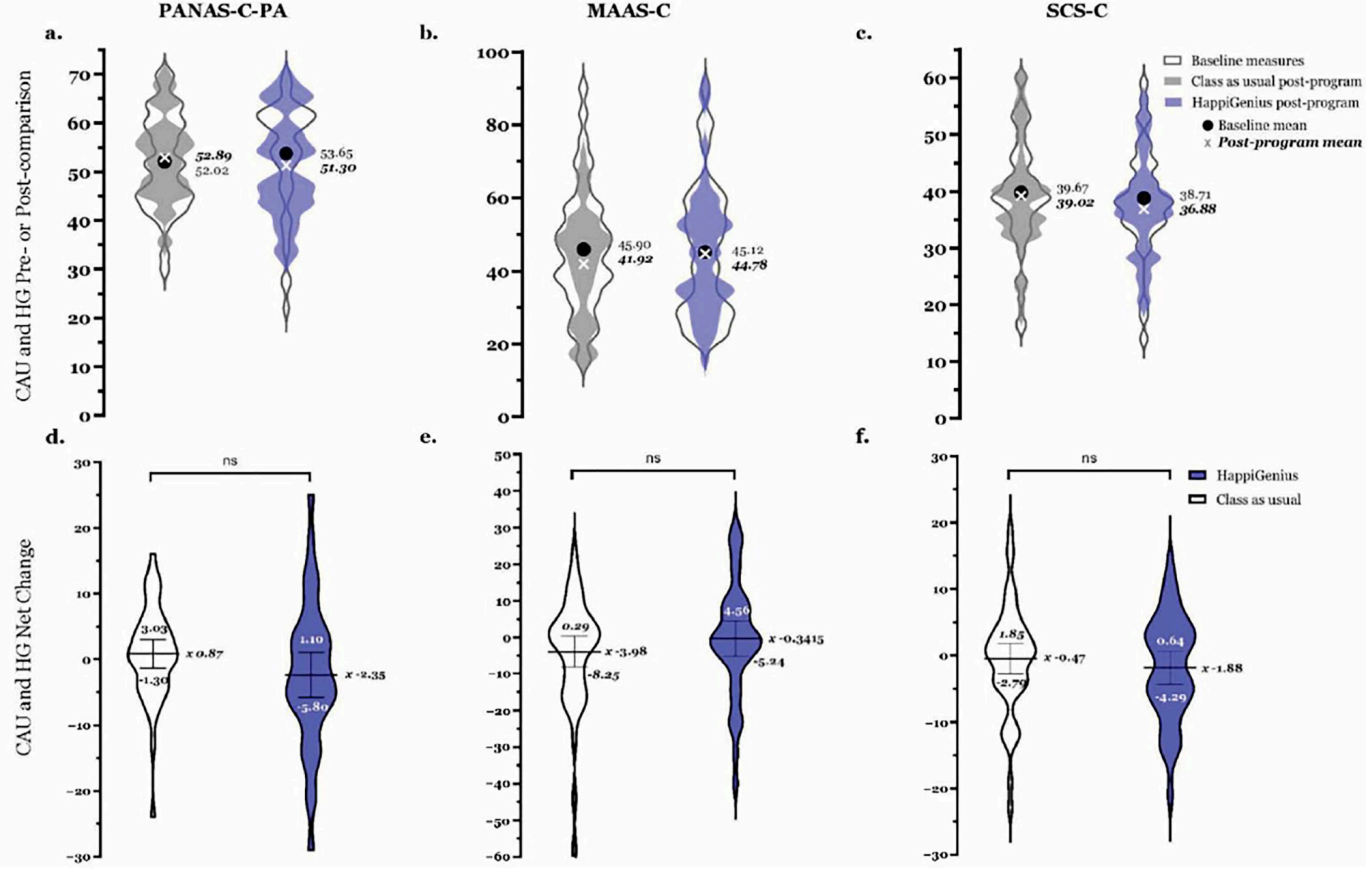
• Illustration of pre–post difference for student-completed measures. (a–c) Distribution of baseline and post-program scores, with the mean (*x*) of baseline (●) and post-program (*x*) scores. Mean and standard deviation were calculated for baseline and post-program survey scores (PANAS-C-PA [*n* HG 40, CAU 45], MAAS-C [*n* HG 41, CAU 49], and SCS-C [*n* HG 41, CAU 50]). For HappiGenius classrooms, the baseline survey score was compared to the post-survey score using multivariable linear regression modeling and found no differences (a) PANAS-C-PA [mean (sd) [pre, post] 53.65 (11.63), 51.30 (10.54) *p* = 0.5241]; (b) MAAS-C [45.12 (18.11), 44.78 (16.87) *p* = 0.7393]; (c) SCS-C [38.71 (9.10), 36.88 (8.66) *p* = 0.2366)]; (d–f) Mean and 95% CI for pre–post changes in HG and CAU classrooms. The mean pre- to post-program change with standard deviation and 95% confidence interval of the mean were calculated as the difference between baseline and post-program survey scores and compared between HG and CAU classrooms. Comparisons were made using multivariable linear regression modeling with the pre–post change (continuous dependent variable), HG or CAU (categorical independent variable), and controlling for school as a covariate. Results were negative, suggesting that there is no difference between the HG and CAU groups in this sample. (a) PANAS-C-PA [mean (sd) [HG, CAU] −2.35 (10.79), 0.87 (7.19) F(1,79) = 2.5, *p* = 0.1155]; (b) MAAS-C [−0.34 (15.53), −3.98 (14.86) F(1,87) = 1.43, *p* = 0.2353]; and (c) SCS-C [−1.88 (7.80), −0.47 (8.16) F(1,85) = 1.18, *p* = 0.2799]. CAU, class as usual; HG, HappiGenius; PANAS-C-PA, Positive and Negative Affect Scale for Children Positive Affect only; MAAS-C, Mindful Attention Awareness Scale for Children; SCS-C, Self-Compassion Scale for Children.

From pre- to post-program, HappiGenius classrooms showed a significant decrease in teacher-reported scores for total difficulties score, internalizing score, and subscales for emotional problems, peer problems, and conduct (Table 1). There was no significant change in teacher-reported prosocial scores, externalizing scores, or the subscale for hyperactivity. Within the HappiGenius classrooms, student self-reports showed an increase in the median mindfulness score [3.00 (−11.50, 16.25)] but not in the mean (Figure 1). However, any observed changes were not statistically significant [F(1,87) = 1.43, *p* = 0.2353] (Figure 1). HappiGenius classrooms did not show significant changes in student measures of positive affect or self-compassion (Figure 1).

Comparison of the pre- to post-survey changes between HappiGenius classrooms and CAU group showed a significant difference favoring HappiGenius classrooms for the internalizing score and peer problems score (Table 1). While teachers anecdotally observed a positive and sustained change, there was no statistically significant difference between HappiGenius classrooms compared to CAU for either the student-reported outcomes or teacher reports of total difficulties, prosocial score, and externalizing score, or the subscales for emotional problems, conduct, and hyperactivity. Analysis of the three-month follow-up survey measures for HappiGenius classrooms showed that the net change from pre- to post-program was statistically similar to the change observed three months post-program, suggesting that the gains made in the fall were maintained at three months post-intervention.

### 3.2. Qualitative results

**Figure 2:**
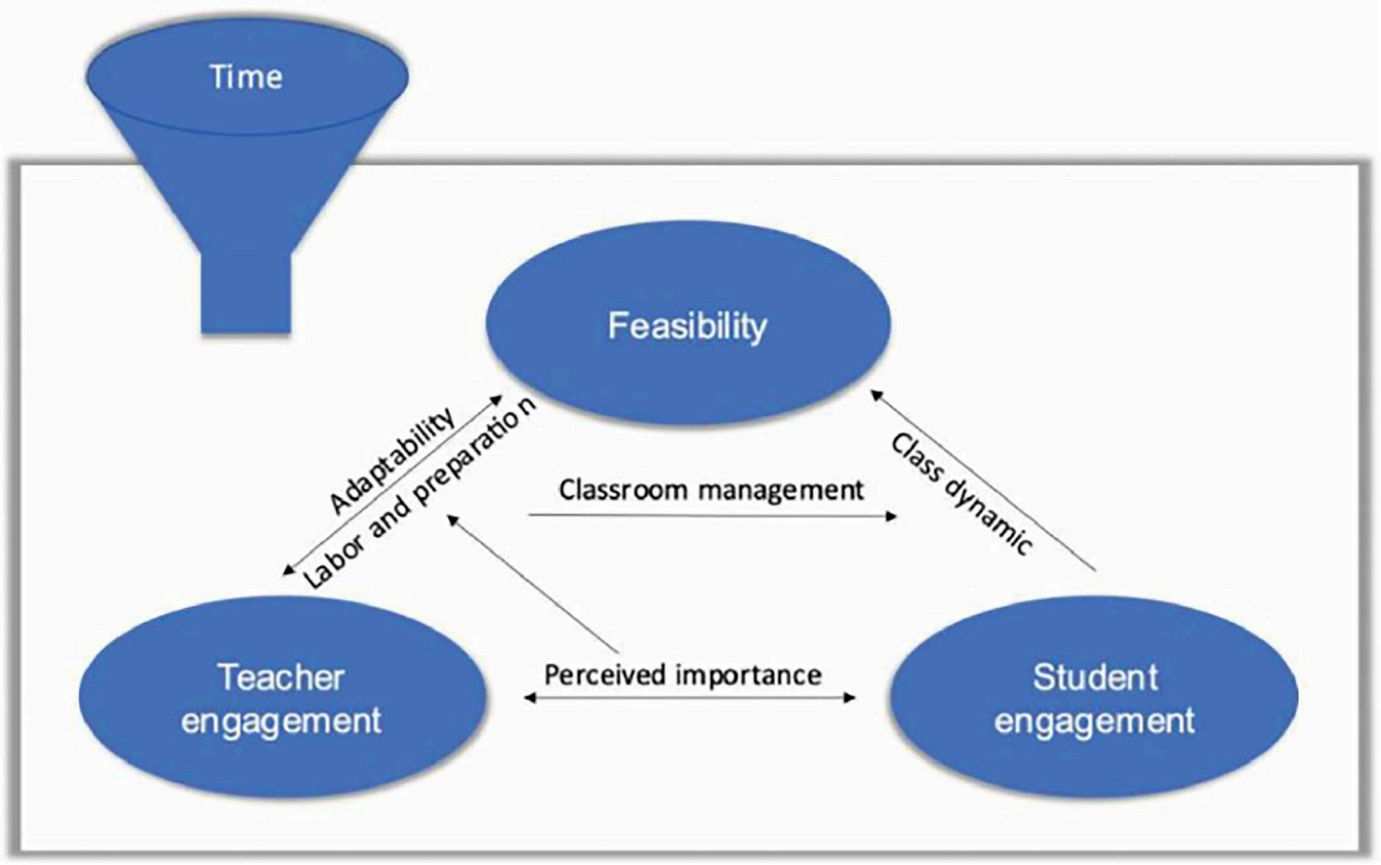
• Subthemes and relationships. The figure illustrates the interactions between concepts and sub-concepts identified in the qualitative analysis. Time, including lesson duration, time of day, and time of year, had overarching effects on all of the concepts and sub-concepts. Three major themes identified in the analysis of teacher interviews illustrated in blue, include teacher engagement, student engagement, and feasibility. Teacher engagement impacted feasibility through the labor and preparation required and willingness to engage in that labor and preparation. The teacher’s perception of importance of the program to their class also appeared to influence the level of labor and preparation they were willing to apply. Teachers who perceived the program materials to be important for the class were able to connect and communicate the relevance of the program for their students, driving student engagement. Teacher’s engagement in classroom management strategies influenced classroom dynamics and had the ability to increase or decrease student engagement depending on the strategies used. Increasing adaptability made the program more feasible for teachers and had the potential to increase their engagement with the program materials.

Comments clustered around three major concepts: teacher engagement, student engagement, and feasibility, all contributing to the overall program acceptability. As illustrated in Figure 2, time was a factor affecting each of these three major concepts (teacher engagement, student engagement, and feasibility). Interconnecting concepts relating teacher engagement to feasibility included labor and preparation and adaptability of the program in the classroom environment. Perceived importance or relevance had a bidirectional relationship with teacher and student engagement, with classroom management serving as an indirect bridge between the two. Student engagement was affected by student types and also related to feasibility through classroom dynamics. Adaptability, labor and preparation, and classroom dynamics all fed back to the feasibility of the program.

#### 3.2.1. Time

The sub-concept time included various dimensions that were barriers or facilitators of teacher engagement, student engagement, and feasibility. These different dimensions are illustrated feeding into the funnel in Figure 2. Teachers from both HappiGenius and CAU classrooms (who received the program in the spring of the same academic year) endorsed the time of year being important for managing student engagement.

So it would be kind of a nice easy thing at the beginning of the year to really incorporate the social–emotional piece in with the classroom learning piece. Like just learning routines and stuff like that.

Specifically, students were thought to be more open to behavioral changes early on in the school year when the classroom dynamic is being established than in the spring when social patterns have become more engrained.

In addition to the time of year, the time of day and the distribution of lessons across the day affected feasibility. “I think one [factor] is just time … kids are, they’ve lost concentration at 2:00 o’clock and they’re here for, you know, another two hours after, which is sad”. Teaching earlier in the school day was a facilitator, while teaching at the end of the school day was a barrier to student engagement. When asked about other challenges, one teacher remarked teaching before lunch or specials (such as art and physical education) was a barrier, while teaching during the short transitions between subjects was a facilitator of student engagement and feasibility. In terms of the distribution of lessons across the day, the ability to adapt the program to fit within the schedule was important to teachers. Teachers who delivered the lessons in a single block (vs. those who broke the lessons into “chunks” delivered across the day) found it affected their students’ ability to stay engaged throughout the lesson. For example, one teacher remarked:

How do you think it was received? I would say half of them liked it, half of them could care less and that would be because it was they had to sit and listen for 30 minutes, 45 minutes and for some of them that’s a long time to sit.

#### 3.2.2. Labor and preparation

Labor and preparation encompassed any work teachers put in to prepare and deliver the lessons. Teacher engagement decreased as teacher labor requirements increased. Program adaptability—the flexibility to adjust the program to fit the needs of the classroom—had a close bidirectional interplay with labor and preparation. In terms of adaptability, some teachers adapted the program by incorporating kinesthetic activities:

Since they’re very active … They talk a lot of things and verbalize a lot of things and then sometimes I’d have them do games where you like ‘stand up if…’ ‘sit down if…’ kind of thing to kind of engage them more kinesthetically.

While incorporating such activities was a classroom management strategy that drove student engagement, it required teachers to engage in the additional labor and preparation. Other teachers “chunked” lessons into smaller pieces delivered between subjects throughout the day rather than in a single block. The ability to chunk lessons was a facilitator of student engagement, but it also required high teacher engagement because of the additional labor and preparation necessary to coordinate and deliver it. Program adaptability and the labor and preparation required of teachers cooperated to drive or inhibit overall feasibility of implementing the program.

Learner level and particularly managing a multilevel classroom increased labor and preparation for teachers delivering the programming. Teachers reported a wide range of skill levels in their students: “We’re from all the way between kindergarten and we’ve got a fifth-grade reader this year. So. I’m trying to get everybody in. It’s a lot”. The dramatic disparity in skill levels was attributed at least in part to fragmented and unsupervised learning in the setting of the COVID-19 pandemic:

… I mean these kids, I think after COVID have really they’ve suffered. Yeah, they’ve suffered with a lot of, you know, I think for some of these third graders, they were in first and 2nd grade during COVID and they were probably home by themselves with an iPad. And it shows, you know.

I’ve noticed a lot this year too with, you know, COVID and having been, you know, out of school for so long that there was really a divide between little my little kids and then my big kids.

The appropriateness of the reading and writing skill level affected teacher and student engagement with the materials in class. Mixed reading and writing skill levels in the same classroom led to difficulty during delivery that was a barrier to full engagement with the program materials. For example,

But for us like I’ve got kids that really struggle with writing and if I were to have them write I have to write everything on the board and wait for them to copy and we would get through about one segment of the lesson. So writing is a challenge.

So then a simple task of, like, even writing three people who love you. If they don’t know how to spell Mom, dad and like, let’s say my name, then it’s the OK, I’m gonna spell it for you.

And then, the next kid. And then, you know. And so that it’s 20 times your spelling random words.

Having such a wide range of student reading and writing skill levels increased labor and preparation for teachers and challenged their abilities to engage students across the classroom. This ultimately had effects on feasibility of the program delivery.

In terms of classroom management strategies, implementing small group and partner work in the activities was considered a facilitator of student engagement and feasibility through classroom dynamic, with student engagement having positive feedback on teachers’ engagement. However, the extra work required to adapt the lessons to small group and partner work was a barrier to using classroom management strategies. When asked about ways they adapted the program to their class to make it more engaging, one teacher described:

Since they’re very active, they’re also very social. So, I did a lot of my own, like turn and talk with your partner things in between. So, in the scenarios when it talks to disappointment, I’d say tell your partner about a time you were disappointed. What did you do? So I changed a lot of it to be like social experiences umm where they explain.

Another teacher did not use partner or small group work but commented:

Or if you- you- if we can incur incorporate more unique ways to do, to get dialogue going and like. The whole group wasn’t, I would make changes to that just myself and like next year, I’m hoping we can sit in like groups. And like have assigned partners and things like that and I just didn’t get that going this time so that they can be ready to share out.

Other classroom management strategies noted by teachers included validation, (providing positive affirmation to students during the lesson), timing and blocking of the lesson delivery as discussed under time, preparing partner and small group work for lesson activities rather than the whole class, and communicating why a lesson was important for the students.

#### 3.2.3. Perceived importance

Perceived importance refers to the individual teachers’ and students’ understanding of the relevance of the program content for their class. The perceived importance of the lessons drove teacher engagement; teachers who considered the content valuable for their students and expressed a belief in the importance of the content were more likely to be engaged with the program and put in additional labor to adapt the program to their classroom needs in a way that enhanced student engagement.

Student engagement was facilitated or inhibited by students’ perceived importance of the program, the individual student type, and the classroom dynamic. For a student to perceive the programming as important required (i) providing rationale to students as to why they are learning a particular lesson or skill and (ii) student buy-in, believing it is important to them. Teachers commented on the perceived importance of the program for their students, such as:

And maybe the only other thing is like more of a link to like we’re watching this video to help you focus on like the, the why, like, why are, why are we watching this? Like, how is that helping the students so the students may be more understand.

One teacher commented on how they used an explanation of the material’s relevance to drive student engagement for certain types of students:

So that was like something we had talked about is why is it important? They’re just at the stage of maturity where they don’t see how it it’s helpful or necessary. So, that was some kids, which I still like had to give them some extra incentives to be serious about it, and to do it.

Another teacher described how the interplay between teacher engagement and student engagement affected program buy-in:

Yeah, like this is we’re working on this because we want you to know how to do some focusing skills. And again [why we’re working on this] wasn’t always clear to me either, but part of that too was not necessarily being as prepared as I could have been to deliver the lessons. It was kind of like we’re gonna do HappiGenius and I’m opening it up and reading through it for the first time kinda as we’re going.

Here the teacher acknowledges how their lack of engagement with lesson preparation affected their own perceived importance of the program components, which in turn could have affected students’ perception of program’s relevance, coming full circle to affect student engagement.

#### 3.2.4. Classroom dynamic

Student engagement and program feasibility were heavily influenced by classroom dynamics. Classroom dynamics were shaped by the time of year, classroom management strategies, student type, and the presence of a guest/volunteer. In terms of the time of year, as previously mentioned, teachers considered fall a better delivery window than spring. This was because of the opportunity to establish classroom norms and boundaries in the fall, to avoid interruptions from state testing in the spring, and to avoid the decrease in classroom attention nearing summer. Classroom management strategies employed to drive student engagement were group/partner work or kinesthetic activities. For example, one teacher modified the star breathing activity:

Like, in any setting or in the hallway or the PE and you’re upset, you can stop and trace your hand and notice how it feels on your skin and that will remind you when you go up on your finger, you inhale. When you go down, you exhale.

Teachers identified different student types that influenced classroom dynamic and ultimately student engagement. Some types of students had a positive effect on classroom dynamics and engagement, while others had a negative effect. For example, some students used humor to draw attention away from the lesson or toward themselves:

Others [students] were just, you know, there was the select few like you that we’re, you know, adding the ‘hmmmmmm’ sound in. And just watching to see if what everyone else is doing and not really focusing on their own breathing.

In reference to multiple-choice questions, “Or they’d like try to be like ohh it’s definitely B and like, try to trick the other kids into thinking it was B and I’m just like you guys”. Still other students approached the lessons with a dismissive or negative attitude:

Umm, so I have one child in particular who’s kind of a leader, like he’s just been made a leader because he’s kind of outgoing and not necessarily in a positive way. So days when he was not here, when we did the lessons, my room was calm and collected. And then when he was here most of my kids still felt that way, but there were definitely some of his friends that were like, I don’t care, I’m over here.

And I mean, I’m not saying that they don’t, maybe internalize it and take it home and do it, but it’s kind of like that “Well, I’m too cool to do it here at school kind of thing”.

So, but I think like some of those more energetic kids that I have, viewed it as being babyish or boring and I don’t know like, just like not as engaging for them. But they’re like very negative about a lot of things.

Lastly, the presence of a guest or volunteer (not used in this run of HappiGenius) was thought to promote student engagement. Teachers remarked “…someone from the outside coming in, like, that has its benefits too because the kids do love it and get excited …” and “If I could make improvements, I would. I do think my kids liked it better when with somebody other than me delivering the instruction”.

#### 3.2.5. Survey feasibility

The teachers universally offered feedback on the student-completed survey instruments. Comments centered around (i) reading and reading comprehension and (ii) the number of answer choices available. In some classrooms, differences in reading and reading comprehension levels led to increased time and effort needed to deliver the surveys.

They [the surveys] took a while, like, to do and then just. I mean, there was just a lot of vocabulary explaining when we did them, so even, you know, just going through them and, but, yeah.

For example, there was confusion among students about the difference between “Happy”, “Joyful”, and “Delighted” on the PANAS-C. The number of answer choices made it difficult to distinguish between options:

I think the survey, … it was too many options for them. … But I think it would be easier for them if we made it simpler, so if it were to be like … sometimes, always, never. Like three choices. Instead of five.

For example, both students and their teachers struggled to gauge the difference between “very frequently” and “somewhat frequently” on the MAAS-C. Teachers were equally perplexed as their students, saying “I can’t remember what exactly was, but there was like one little word of a difference. And I was like, I can’t even tell you the difference between what this one is and that one”.

## 4. Discussion

In comparison to control classrooms, HappiGenius classrooms showed a statistically significant improvement in the internalizing and peer problems subscales but not in the other teacher- or student-reported measures. The present study found a significant decrease from pre- to post-HappiGenius programming on teacher reports for overall difficulties, internalizing tendencies, and subscales for emotional problems, peer problems, and conduct but not for hyperactivity or prosocial scores. These findings are similar to those of the pilot study, with the exception of prosocial scores, which showed improvement in the pilot but not in this study [27].

Internalizing behaviors are directed inward and are reflective of internal emotional states. Internalizing behaviors have the potential to develop into internalizing disorders, such as depression, anxiety, and somatic symptoms [11]. In Western cultures, internalizing disorders are often associated with punishment and are linked to behavioral inhibition, which involves retreating inward in response to difficult situations [11]. This can lead to a myriad of effects, one of which is isolation and problems connecting to and relating with peers [36, 37]. In SEL, externalizing behaviors have historically been a common target for programming [17]. Studies of parents and teachers generally find that they express more concerns about externalizing behaviors, likely because, as the name suggests, these externalizing behaviors are readily observable and affect those around us. Externalizing and internalizing behaviors seem to have a complex bidirectional relationship. For example, children with externalizing features can also have internalizing issues or children with internalizing symptoms can have negative outward effects on peers and family [11]. It is for these reasons, and due to the increasing awareness of the areas of anxiety, depression, and death by suicide among young people, that there has been a call for preventative programs, including CASEL’s establishment of internalizing behaviors as an important independent program target [11, 17]. In our study, the significant improvement in internalizing and peer problems for HappiGenius classrooms compared to CAU was complemented by teachers’ thoughts on ideal timing of the program. Teachers suggested that delivering the program in the fall facilitated development of a positive classroom dynamic and set important standards for appropriate classroom behaviors; reduction of peer problems may be one of the mechanisms by which this happens.

Negative results of the student measures for positive affect, self-compassion, and mindfulness also differed from what was expected based on the pilot study, where positive affect and self-compassion improved significantly pre- to post-program. Surprisingly, despite addressing the issues identified in the pilot study, we did not observe a change in mindfulness and hyperactivity. Changes in the program delivery included teaching over the course of 12 weeks as the program was originally designed, compared to an abbreviated six-week delivery in the pilot study. The new delivery was given at the beginning of the school year instead of at the end, near summer, and was delivered by the classroom teacher rather than an outside delivery agent [27]. As a mindfulness-based program, we expected to see the most consistent changes in these areas. At the time of the pilot study, it was thought that the novelty of an outside agent delivering the program might have contributed to hyperactivity-type behaviors. Interestingly, qualitative feedback from the teachers in this study highlighted how students find outside instructors novel and thus may pay greater attention, leading to higher gains from the HappiGenius program. Another variable identified in the qualitative feedback that was not controlled in the study design was the time of day the program was delivered. Teachers suggested avoiding certain times of day for program delivery, including before lunch or before the end of the school day. However, our program study is not isolated in finding conflicting or unexpected results related to student attention. For example, another mindfulness-based program showed positive results in one study but negative results in another (Schonert-Reichl et al. [21] vs. de Carvalho et al. [38]).

Additionally, it is worth mentioning a recently posited hypothesis related to mindfulness teaching in the school setting. This study found an increased risk of depression and diminished well-being following a different mindfulness training program in a subset of students who scored high on a measure of depressive symptoms [the Center for Epidemiological Studies Depression Scale (CES-D)] [39]. Though not yet tested in the literature, the authors posited that teaching mindfulness could bring attention to upsetting thoughts and feelings, and without adequately enhancing resiliency first, could lead to detrimental effects for certain subgroups. This could be one explanation for the observed increase in mindfulness and the decrease in positive affect and self-compassion in summary statistics and correlation analyses. However, HappiGenius is a mindfulness-based SEL program that includes evidence-based exercises to build resiliency, compared to a mindfulness training curriculum. Considering the co-delivery of mindfulness and resiliency teaching, another possibility is that (1) mindfulness develops faster than resilience, leading to a dip in positive affect and self-compassion, or (2) there was a heavy influence of social factors that are more difficult to be resilient against. Other key differences between our sample and the sample studied by Montero-Marin et al. [39] include student age and study design. Our students aged eight to nine are at an earlier developmental stage than early adolescents aged 11–13 [39]. Our study was an effectiveness (versus efficacy) design and analyzed effects on the entire group overall, without exclusion based on existing difficulties or diagnosed psychopathology. This difference may affect baseline levels and room for growth. In the Montero-Marin et al. [39] study, they assessed subgroups sorted by risk of depression. When the same dataset was used to assess overall changes for all students without stratifying by baseline risk of depression, there was no significant risk to depression or well-being following the program [39]. This opens opportunities for future lines of study, including investigating outcomes based on the order and timing of delivery of resilience and mindfulness components and conducting longer-term follow-up to determine whether measures rebound after an initial dip.

There were benefits to student behavior reported in the qualitative analysis besides internalizing and peer problems. Improving internalizing issues can positively affect behavior and peer relationships, but there are also several other factors specific to the study design and implementation that could have masked quantitative outcomes that were observable to teachers in the classroom. As previously mentioned, we employed an effectiveness design rather than an efficacy design in this study. A common underlying assumption in the efficacy-based paradigm is to control as many potential confounders as possible to isolate the effects of a single intervention on an outcome [40]. While this approach increases the internal validity of a study, it often limits its applicability to the real world. In contrast to efficacy, an effectiveness paradigm seeks to evaluate programs in the real world, with all its variability, to determine whether there is an impact. While the outcomes of an effectiveness study may appear smaller, it may ultimately confer a greater benefit when disseminated because it is already proven to be able to reach the population outside of a tightly controlled research environment [40]. Other than the study design, teachers reported difficulties with the student survey. Traditionally, we see other studies that have targeted younger children in pre-K or kindergarten relying on teacher and parent reports, while self-reports are more commonly used in older children (fifth grade plus). Given the intermediate developmental stage as third graders, we considered important to include both outsider measures (teacher) and self-measures to have insights to students’ internal processing and experience. While the surveys were studied and validated in the target age group, teachers noted a wide variety of reading and writing skill levels within each classroom, largely attributed to differences in children’s experience of education during COVID-19 shutdowns. One major point of feedback that came up with multiple teachers was the Likert scale. The surveys used Likert scales of 5–7 points, but many children were reported to struggle with making decisions between options like very frequently and somewhat frequently. A teacher suggested that three-item options may be more developmentally appropriate. Even outside of the COVID-19 setting, the use of 3 or 4-item Likert scales with or without visual aids has been supported by other research literature over 2- or 5-point scales [41–43]. This should be an important consideration for researchers aiming to design surveys and complete studies in this age group, especially as we increasingly focused on internalizing behaviors, which might be better reported by students than external observers.

The study provided data to inform practical recommendations for educators engaged in SEL curriculum delivery. These are also important considerations for researchers to consider as possible variables in intervention study design. Based on qualitative feedback and backed by evidence of biological influences on attention [44], timing strategies for optimizing delivery of a program for improved student focus and engagement include:

- delivery of social–emotional programming early in the school year rather than late to maximize benefit for classroom;
- avoiding certain times of day when attention wanes, such as just before lunch or at the end of the school day;
- chunking lessons into shorter segments delivered at multiple times throughout the day, rather than in one continuous lesson;
- selecting timelines with awareness of outside interruptions such as state testing.

In addition, teachers identified several classroom management strategies benefiting students and the overall feasibility of incorporating the program. These might be considered for an educator’s use in the classroom or incorporated into SEL program designs and implementation research:

- taking time to communicate the relevance of a lesson to students (self-determination theory further described in Shankland et al. [22]),
- utilizing partner-based work and discussions, rather than whole class,
- incorporating kinesthetic components,
- providing validation and positive affirmation to students during the lesson.

As an additional note to program developers and researchers, just as it is important for teachers to communicate the relevance of an intervention for their class, it is also important that teachers believe the content to be important for their students. Perhaps, effectiveness might be further enhanced if teachers participate in similar, though age-appropriate, training prior to program delivery in their classrooms.

Teachers reflected that the HappiGenius curriculum was adaptable within and between classrooms and valued the flexibility to fit the program to the particular needs of their classrooms. However, the extra labor required of the teacher to make those changes can be a barrier. Programs such as HappiGenius would likely benefit from differentiating the lessons for the teachers using strategies such as those outlined above. From a researcher’s perspective, one concern is that variation in delivery between classrooms in the real world introduces confounding variables. There may be a happy medium where iterative changes are made to the program based on rigorously assessing and evaluating feedback of effectiveness studies. Incorporating changes informed by feedback and study outcomes into the core curriculum as best practices could help decrease some of the additional labor that teachers took on adapting the curriculum to their classrooms. Reducing teacher labor was noted to increase teacher engagement and drive feasibility. From the researcher standpoint, shoring up some of the differences in delivery between classrooms can naturally increase the internal validity of the study.

### 4.1. Limitations

The study was conducted in a school district located in a midsized city in the Midwestern USA, and the generalizability of the study to other geographic locations is limited. Sex, gender, age, and ethnicity data were available at the school level but not at the individual level. Sex and gender have reported differences between some SEL targets and measures, as well as endpoints like anxiety [7, 11]. These data restrictions prevented a more indepth analysis and comparison. At the level of the schools, students were primarily non-Hispanic White, followed by Black, Hispanic/Latinx, Asian, and Native American (Table S1, Supplementary material). The generalizability to other communities is unknown. As explained in the discussion, there are benefits and drawbacks to an effectiveness study design. It is less likely to observe large changes in effectiveness studies. However, we can be more confident that the significant changes we do observe will translate into other real-world environments and confer similar benefits. One of the challenges in researching within the study setting in our partner school district is the rapid adoption of several different approaches to SEL. For this reason, the longitudinal study was selected to cover one academic year to avoid additional noise in the data. At the time of the study, Second Step was in use district-wide for a number of years, including in the schools participating in the HappiGenius study. Second Step is an established social and emotional learning program with a large evidence base, including improving emotional problems, hyperactivity, and skills learning in students starting out with lower baseline SEL competencies [45]. Since both HappiGenius classrooms and CAU received the Second Step curriculum, we maintained this as a baseline characteristic, though it could have masked some of the effects in both groups. In terms of survey methodology, there are several drawbacks to the repeated measures design. Individuals who were missing one or more items on analysis were excluded from study for that measure. Given the reading comprehension difficulties noted by teachers in the study, it is possible that bias was introduced to the student measures if those individuals did not complete the surveys and were systematically excluded. In addition, participants filling out the same measures each time, even though they were separated by several months, introduce the possibility of order effects. Finally, the study had a very specific focus on social–emotional learning programs delivered in the elementary school setting. There are other spheres of influence not included in the study that are highly relevant to children’s well-being and psychosocial health. These include the influence of family, especially parents; social conditions, including socioeconomic status and diversity; personal crises; and exposure to trauma, among others [1, 9, 46].

## 5. Conclusions

Students receiving HappiGenius showed significant improvement from baseline in overall difficulties, internalizing tendencies, and subscales for emotional problems, peer problems, and conduct. HappiGenius showed a significant change compared to CAU only for internalizing symptoms and specifically peer problems. Complimenting the finding of improvement in peer problems, teacher comments endorsed HappiGenius as an early intervention to establish classroom dynamics and behavioral norms at the start of the school year before problem behaviors develop. Teachers suggested best practices in the areas of delivery timing and classroom management. In program design, the competing values of program adaptability and implementation integrity need to be balanced, along with considerations for the labor and preparation necessary to deliver the program.

## Supplementary Material

Supplementary Materials

## Figures and Tables

**Table 1:** • HappiGenius pre–post differences and comparisons to CAU for teacher-completed survey measures

Scale (n HG, CAU)	HappiGenius			Class as usual		Pre–post change		
	Pre	Post	*p*	Pre	Post	HG	CAU	*p*
SDQ Total (64.48)	28 (22.25, 32.75)	26 (21, 32.75)	0.0010*	25 (22, 31)	25.5 (21, 33)	−1.5 (−3, 0.75)	0.00 (−2, 1)	0.1566
Internalizing	13 (12, 16)	13 (12, 15.75)*	<0.0001*	13 (12, 16)	13 (12, 15.75)	0.00 (−2, 0.00)	0.00 (−2, 1.)	0.0389*
Peer	7 (7, 9.75)	7 (7, 8)*	0.0008*	7 (7, 8)	7 (7, 8.00)	0.00 (−1, 0.00)	0.00 (0.00, 0.00)	0.0284*
Emotion	5 (5, 7.75)	6 (5, 7)*	0.0033*	5 (5, 8)	5 (5, 7.75)	0.00 (−1.5, 0.00)	0.00 (−1, 0.00)	0.5646
Externalizing	12.5 (10, 18)	13.00 (10, 18.75)	0.1340	13 (10, 16)	13 (10, 18)	0.00 (−2, 2.5)	1 (−2, 6.25)	0.1457
Conduct	5 (5, 7)	5 (5, 7.00)*	0.0110*	5 (5, 6)	5 (5, 7)	0.00 (−1, 0.00)	0.00 (0.00, 0.00)	0.1151
Hyperactivity	7 (5, 10.75)	8 (5, 11.75)	0.3980	8 (5, 10)	7.5 (5, 11)	0.00 (−1, 0.5)	0.00 (−1, 1)	0.8254
Prosocial	12 (8.25, 15)	14.50 (11, 15)	0.4335	15 (12.25, 15)	15 (11.25, 15)	0.00 (−1, 1)	0.00 (−1, 0.00)	0.2331

The median and interquartile range (SDQ) scores were calculated for baseline survey scores, post-program survey scores, and the pre–post change. For HappiGenius classrooms, the baseline survey score was compared to the post-survey score using the Wilcoxon matched-pairs signed-rank test. There were significant positive changes from baseline for total difficulties, the internalizing subscale, the emotion subscale, the peer problems subscale, and the conduct subscale. The pre–post change, calculated as the difference between baseline and post-program survey scores, was compared between HappiGenius and CAU classrooms. Mann–Whitney tests were used for comparisons. Compared to CAU, HappiGenius classrooms showed significant positive changes on the internalizing subscale and the peer problems subscale.

* denotes statistical significance. HG, HappiGenius; CAU, class as usual; SDQ-T, Strengths and Difficulties Questionnaire – Teacher completed.

## Data Availability

Data supporting these findings are available within the article, at https://doi.org/10.20935/MHealthWellB7308, or upon request.

## References

[1] Gray P, Lancy DF, Bjorklund DF. Decline in independent activity as a cause of decline in children’s mental well-being: summary of the evidence. J Pediatr. 2023;260:113352. doi: 10.1016/j.jpeds.2023.02.004 36841510

[2] Twenge JM, Gentile B, DeWall CN, Ma D, Lacefield K, Schurtz DR. Birth cohort increases in psychopathology among young Americans, 1938–2007: a cross-temporal meta-analysis of the MMPI. Clin Psychol Rev. 2010;30(2):145–54. doi: 10.1016/j.cpr.2009.10.005 19945203

[3] Bommersbach TJ, McKean AJ, Olfson M, Rhee TG. National trends in mental health-related emergency department visits among youth, 2011–2020. JAMA. 2023;329(17):1469–77. doi: 10.1001/jama.2023.4809 37129655 PMC10155071

[4] Brannen DE, Wynn S, Shuster J, Howell M. Pandemic isolation and mental health among children. Disaster Med Public Health Prep. 2023;17:e353. doi: 10.1017/dmp.2023.7 36628622 PMC10019926

[5] Knier K, Weinman A, Mullan A, Cain M, Hevesi S, Bellam-konda VR. Association between school learning models and psychological and social health visits to the emergency room. J Am Coll Emerg Physicians Open. 2024;5(2):e13157. doi: 10.1002/emp2.13157 38634074 PMC11021855

[6] Kovacs B, Caplan N, Grob S, King M. Social networks and loneliness during the COVID-19 pandemic. Socius. 2021;7:2378023120985254. doi: 10.1177/2378023120985254

[7] Meherali S, Punjani N, Louie-Poon S, Abdul Rahim K, Das JK, Salam RA, Mental health of children and adolescents amidst COVID-19 and past pandemics: a rapid systematic review. Int J Environ Res Public Health. 2021; 18(7):3432. doi: 10.3390/ijerph18073432 33810225 PMC8038056

[8] O’Sullivan K, Clark S, McGrane A, Rock N, Burke L, Boyle N, A qualitative study of child and adolescent mental health during the COVID-19 pandemic in Ireland. Int J Environ Res Public Health. 2021;18(3):1062. doi: 10.3390/ijerph18031062 33504101 PMC7908364

[9] Boat TF, Kelleher KJ. Fostering healthy mental, emotional, and behavioral development in child health care. JAMA Pediatr. 2020;174(8):745–6. doi: 10.1001/jamapediatrics.2020.1485 32568384

[10] Hudson JL, Minihan S, Chen W, Carl T, Fu M, Tully L, Interventions for young children’s mental health: a review of reviews. Clin Child Fam Psychol Rev. 2023;26(3):593–641. doi: 10.1007/s10567-023-00443-6 37488453 PMC10465658

[11] Liu J, Chen X, Lewis G. Childhood internalizing behaviour: analysis and implications. J Psychiatr Ment Health Nurs. 2011;18(10):884–94. doi: 10.1111/j.1365-2850.2011.01743.x 22070805 PMC5675073

[12] National Academies of Sciences, Engineering, and Medicine; Division of Behavioral and Social Sciences and Education; Board on Children, Youth, and Families; Committee on Fostering Healthy Mental, Emotional, and Behavioral Development Among Children and Youth. The National Academies Collection: Reports funded by National Institutes of Health. Fostering healthy mental, emotional, and behavioral development in children and youth: a national agenda. Washington (DC): National Academies Press; 2019. doi: 10.17226/25201 31869055

[13] Hwang WJJ, Hyun Hee. Impact of mental health on wellness in adult workers. Front Public Health. 2021;9:743344. doi: 10.3389/fpubh.2021.743344 34976913 PMC8716594

[14] Miller G, Foster L. A brief summary of holistic wellness literature. J Holist Healthcare. 2010;7(1):4–8.

[15] Ryff CD, Singer B. The contours of positive human health. Psychol Inq. 1998;9(1):1. doi: 10.1207/s15327965pli0901_1

[16] Smith TSJ, Reid L. Which ‘being’ in wellbeing? Ontology, wellness and the geographies of happiness. Prog Hum Geogr. 2017;42(6):807–29. doi: 10.1177/0309132517717100

[17] Skoog-Hoffman A, Ackerman C, Boyle A, Schwartz H, Williams B, Jagers R, Evidence-based social and emotional learning programs: CASEL criteria updates and rationale; 2020. p. 1–42. Available from: https://casel.org/wp-content/uploads/2021/01/11_CASEL-ProgramCriteria-Rationale.pdf

[18] Durlak JA, Weissberg RP, Dymnicki AB, Taylor RD, Schellinger KB. The impact of enhancing students’ social and emotional learning: a meta-analysis of school-based universal interventions. Child Dev. 2011;82(1):405–32. doi: 10.1111/j.1467-8624.2010.01564.x 21291449

[19] Hawkins JD, Kosterman R, Catalano RF, Hill KG, Abbott RD. Promoting positive adult functioning through social development intervention in childhood: long-term effects from the Seattle Social Development Project. Arch Pediatr Adolesc Med. 2005;159(1):25–31. doi: 10.1001/archpedi.159.1.25 15630054

[20] Hawkins JD, Kosterman R, Catalano RF, Hill KG, Abbott RD. Effects of social development intervention in childhood 15 years later. Arch Pediatr Adolesc Med. 2008;162(12):1133–41. doi: 10.1001/archpedi.162.12.1133 19047540 PMC2593733

[21] Schonert-Reichl KA, Oberle E, Lawlor MS, Abbott D, Thomson K, Oberlander TF, Enhancing cognitive and social-emotional development through a simple-to-administer mindfulness-based school program for elementary school children: a randomized controlled trial. Dev Psychol. 2015;51(1):52–66. doi: 10.1037/a0038454 25546595 PMC4323355

[22] Shankland R, Haag P, Tessier D, Buchs C, El-Jor C, Mazza S. Review of the effects of social and emotional learning on mental health and academic outcomes: the role of teacher training and supportive interactions. J Epidemiol Popul Health. 2024;72(3):202750. doi: 10.1016/j.jeph.2024.202750 38848636

[23] Broderick PC, Frank JL. Learning to BREATHE: an intervention to foster mindfulness in adolescence. New Dir Youth Dev. 2014;2014(142):31–44. doi: 10.1002/yd.20095 25100493

[24] Op’t Eynde P, Turner JE. Focusing on the complexity of emotion issues in academic learning: a dynamical component systems approach. Educ Psychol Rev. 2006; 18(4):361–76. doi: 10.1007/s10648-006-9031-2

[25] Lawlor MS. Mindfulness and social emotional learning (SEL): a conceptual framework. Handbook of mindfulness in education. mindfulness in behavioral health. New York, NY: Springer; 2016. p. 65–80. doi: 10.1007/978-1-4939-3506-2_5

[26] Dunning DL, Griffiths K, Kuyken W, Crane C, Foulkes L, Parker J, Research review: the effects of mindfulness-based interventions on cognition and mental health in children and adolescents - a meta-analysis of randomized controlled trials. J Child Psychol Psychiatry. 2019;60(3): 244–58. doi: 10.1111/jcpp.12980 30345511 PMC6546608

[27] Knier K, Sood G, Ruffin W, Arroyo J, Sabharwal A, Bostwick M, Implementation of a novel social-emotional learning program to advance integration of wellness in education practice. J Adv Educ Pract. 2023;4(1):article 5. Available from: https://openriver.winona.edu/jaep/vol4/iss1/5 PMC12453881 40988697

[28] Porter B, Oyanadel C, Sáez-Delgado F, Andaur A, Peñate W. Systematic review of mindfulness-based interventions in child-adolescent population: a developmental perspective. Eur J Investig Health Psychol Educ. 2022;12(8):1220–43. doi: 10.3390/ejihpe12080085 PMC9407079 36005234

[29] HappiGenius. Become a HappiGenius instructor;2021 [cited 2024 July]. Available from: https://www.happigenius.com

[30] Fiorelli JA. The differential prediction of positive and negative affect in play and in daily life in children. Cleveland (OH): Case Western Reserve University; 2015. Available from: https://dl.acm.org/doi/10.5555/AAI28079025

[31] Hughes AA, Kendall PC. Psychometric properties of the positive and negative affect scale for children (PANAS-C) in children with anxiety disorders. Child Psychiatry Hum Dev. 2009;40(3):343–52. doi: 10.1007/s10578-009-0130-4 19142724

[32] Laurent J, Catanzaro SJ, Joiner TE Jr, Rudolph KD, Potter KI, Lambert S, A measure of positive and negative affect for children: scale development and preliminary validation. Psychol Assess. 1999;11(3):326–38. doi: 10.1037/1040-3590.11.3.326

[33] Sutton E, Schonert-Reichl KA, Wu AD, Lawlor MS. Evaluating the reliability and validity of the self-compassion scale short form adapted for children ages 8–12. Child Indic Res. 2018;11(4):1217–36. doi: 10.1007/s12187-017-9470-y

[34] Stone LL, Otten R, Engels RC, Vermulst AA, Janssens JM. Psychometric properties of the parent and teacher versions of the strengths and difficulties questionnaire for 4- to 12-year-olds: a review. Clin Child Fam Psychol Rev. 2010;13 (3):254–74. doi: 10.1007/s10567-010-0071-2 20589428 PMC2919684

[35] van den Heuvel M, Jansen D, Stewart RE, Smits-Engelsman BCM, Reijneveld SA, Flapper BCT. How reliable and valid is the teacher version of the Strengths and Difficulties Questionnaire in primary school children? PLoS One. 2017; 12(4):e0176605. doi: 10.1371/journal.pone.0176605 28453573 PMC5409073

[36] Rubin KH, Coplan RJ, Bowker JC. Social withdrawal in childhood. Annu Rev Psychol. 2009;60:141–71. doi: 10.1146/annurev.psych.60.110707.163642 18851686 PMC3800115

[37] Schwartz D, McFadyen-Ketchum S, Dodge KA, Pettit GS, Bates JE. Early behavior problems as a predictor of later peer group victimization: moderators and mediators in the pathways of social risk. J Abnorm Child Psychol. 1999;27 (3):191–201. doi: 10.1023/a:1021948206165 10438185 PMC2761646

[38] de Carvalho JS, Pinto AM, & Marôco J (2017). Results of a mindfulness-based social-emotional learning program on Portuguese elementary students and teachers: A quasi-experimental study. Mindfulness, 8(2), 337–350. doi: 10.1007/s12671-016-0603-z

[39] Montero-Marin J, Allwood M, Ball S, Crane C, De Wilde K, Hinze V, School-based mindfulness training in early adolescence: what works, for whom and how in the MYRIAD trial? Evid Based Ment Health. 2022;25(3):117–24. doi: 10.1136/ebmental-2022-300439 35820993 PMC9340034

[40] Glasgow RE, Vogt TM, Boles SM. Evaluating the public health impact of health promotion interventions: the RE-AIM framework. Am J Public Health. 1999;89(9):1322–7. doi: 10.2105/ajph.89.9.1322 10474547 PMC1508772

[41] Mellor D, Moore KA. The use of Likert scales with children. J Pediatr Psychol. 2013;39(3):369–79. doi: 10.1093/jpepsy/jst079 24163438

[42] Alan Ü, Atalay Kabasakal K. Effect of number of response options on the psychometric properties of Likert-type scales used with children. Stud Educ Eval. 2020;66:100895. doi: 10.1016/j.stueduc.2020.100895

[43] Arsiwala T, Afroz N, Kordy K, Naujoks C, Patalano F. Measuring what matters for children: a systematic review of frequently used pediatric generic PRO instruments. Ther Innov Regul Sci. 2021;55(5):1082–95. doi: 10.1007/s43441-021-00311-x 34142363 PMC8332594

[44] Wile AJ, Shouppe GA. Does time-of-day of instruction impact class achievement? Perspect Learn. 2011;12(1):9. Available from: https://csuepress.columbusstate.edu/pil/vol12/iss1/9

[45] Low S, Cook CR, Smolkowski K, Buntain-Ricklefs J. Promoting social–emotional competence: an evaluation of the elementary version of Second Step^®^. J Sch Psychol. 2015; 53(6):463–77. doi: 10.1016/j.jsp.2015.09.002 26563599

[46] Bitsko RH, Holbrook JR, Ghandour RM, Blumberg SJ, Visser SN, Perou R, Epidemiology and impact of health care provider-diagnosed anxiety and depression among US children. J Dev Behav Pediatr. 2018;39(5):395–403. doi: 10.1097/DBP.0000000000000571 29688990 PMC6003874

