# Supplementary material for: Social–emotional learning program: a community-based case-controlled study
Source: Acad Ment Health Well Being. Author manuscript; Available in PMC 2025 Apr 21. (PMC12011368; doi:10.20935/mhealthwellb7308)
Supplement: Supplementary Materials [file NIHMS2024539-supplement-Supplementary_Materials.pdf]

Supplementary materials

Table S1 • School and district demographic characteristics

**Participating schools**	**A**	**B**	**C**	**D**	**District**
Total enrollment (*n*)	398	405	461	653	7,999
Third graders	62	65	54	96	1,224
Sex (%)					
Female	48	53	46	53	48
Race/Ethnicity, *n* (%)					
White	168 (42)	121 (30)	333 (72)	207 (32)	4,324 (54)
Black or African American	89 (22)	88 (22)	34 (7)	172 (26)	1,279 (16)
Asian	45 (11)	28 (7)	36 (8)	68 (10)	686 (9)
American Indian/Alaskan Native	0 (0)	3 (<1)	0 (0)	3 (<1)	21 (<1)
Native Hawaiian/Pacific Islander	0 (0)	0 (0)	0 (0)	3 (<1)	9 (<1)
Identified as two or more races	48 (12)	65 (16)	37 (8)	72 (11)	726 (9)
Hispanic or LatinX, any race	48 (12)	100 (25)	21 (5)	128 (20)	954 (12)
Free or reduced lunch eligible	210 (53)	292 (72)	65 (14)	392 (60)	

The details for each school and the school district were sourced from the National Center for Education Statistics' Common Core of Data public school data for the 2021–2022 academic year.

Ia. Variables and measures

Positive Affect Scale for Children (PANAS-C-PA): The PANAS-C-PA is one section of the Positive and Negative Affect Scale (PANAS), a self-report inventory of the frequency of positive and negative emotions over the last two weeks [1]. The PANAS adapted for children (PANAS-C) was developed and tested in a clinical sample of *n* = 358 children aged 9–14 years, with high reliability (Cronbach's alpha = 0.89) that has been replicated in other studies with student participants [1]. We use the positive affect scale only and refer to it as PANAS-C-PA. Our study replicated the high reliability with the pre-survey Cronbach's alpha = 0.872 and post-survey Cronbach's alpha = 0.837.

Self-Compassion Scale for Children (SCS-C): The SCS-C is a self-report measure for self-compassion, a trait that includes (1) self-kindness, (2) common humanity vs. a sense of isolation, and (3) mindfulness of current experience. The SCS-C has been validated in a student sample of *n* = 382 children aged 8–12 with high reliability (Cronbach's alpha = 0.81–0.83) [2]. In our study, reliability measures were lower with the pre-survey Cronbach's alpha = 0.631 and post-survey Cronbach's alpha = 0.571. The SCS-C has demonstrated convergent validity with a strong positive relationship to MAAS-C scores and constructs including self-concept, positive affect, and prosocial goals. The SCS-C has appropriate divergent validity from depression and anxiety scores [2].

Mindful Attentive Awareness Scale for Children (MAAS-C): The MAAS-C is a self-report of attention to the current experience from moment to moment. The MAAS-C is a 15-item survey with a 6-point Likert response scale. The MAAS-C measures mindfulness as a unidimensional trait, shows appropriate discriminant and divergent validity in reference to other dimensions of self-awareness and social-emotional wellbeing, and has adequate internal consistency in a non-clinical sample (0.84 in *n* = 593 students aged 12–15 and 0.84 in *n* = 286 students aged 9–13, respectively) [3]. Reliability was high on pre-survey (0.899) and post-survey measures (0.894) for this study.

Strengths and Difficulties Questionnaire—Teacher completed (SDQ-T): The SDQ-T is a well-validated, 25-item, teacher-completed measure of student attention, sociality, and behavioral health [4, 5]. The teacher rates 25 items for each student on a 3-point Likert-type scale. The output of the SDQ-T includes a total difficulties score with four subscales (emotional problems (internalizing), conduct problems (externalizing), hyperactivity/ inattention (externalizing), and peer problems (internalizing)) and a prosocial score. Reliability for the present study was high, with the pre-survey Cronbach's alpha = 0.883 and the post-survey Cronbach's alpha = 0.916.

Table S2 • Preselected correlation analysis results

Pre (rs, *p*-value)	PANAS	MAAS	SCS	SDQ total difficulties
PANAS-C-PA		(−)0.2107, 0.0192	0.4642, <0.0001	(−)0.2508, 0.0066
MAAS-C			(−)0.3963, <0.0001	0.1913, 0.0276
SCS-C				(−)0.1112, 0.1329

Note: The shading denotes those comparisons that achieved statistical significance. The gray is to indicate redundant/ comparisons not made. Significant correlations were identified between MAAS-C and PANAS-C-PA; SCS-C and PANAS-C-PA; and SCS-C and MAAS-C, which were reassuring for construct validity. MAAS-C and PANAS-C-PA each independently correlated with SDQ total difficulties score, indicating some agreement between student- and teacher-reported attributes. There was no significant correlation between SDQ total difficulties score and SCS-C. PANAS-C-PA, Positive and Negative Affect Scale for Children—Positive Affect only; MAAS-C, Mindful Attentive Awareness Scale for Children; SCS-C, Self-Compassion Scale for Children; SDQ, Strengths and Difficulties Questionnaire (teacher completed).

Ib. Interview guide for semi-structured teacher interviews

My goal for doing this interview is to try to better understand your experience with HappiGenius in your classroom by asking you to describe how you see things. Do you have any questions before we begin?

1. Can you describe what an average lesson in HappiGenius looked like in your classroom?
   a. Did you incorporate breaks throughout the lessons? (How many/how often?)
2. What are your thoughts about the HappiGenius program you delivered this term?
   a. What changes would you make to improve the program? Why?
   b. What problems did you come across with the program content or delivery?
3. In your own opinion, was the HappiGenius program worthwhile for your students? Would you run the program again? (Why?)
4. How do you think HappiGenius was received by your students? How engaged were your students with the lessons?
   a. Did you find any of the lessons or activities particularly engaging for your students?
   b. Why do you think these lessons were especially engaging?
5. Did you notice any change in your classroom dynamic?
6. Did you notice your students using any of the skills they learned outside of HappiGenius lessons?
7. What are your thoughts on using surveys to evaluate student outcomes for the HappiGenius program?
   a. Are there changes being missed by the survey assessments?
   b. How do you think we can best capture these other changes?

Are there topics we should explore that I haven’t asked about?

Table S3 • Baseline comparisons for student- and teacher-completed survey measures

**Measure**<br>**(*n* HappiGenius, Class as usual)**	**HappiGenius**<br>**(Median, IQR)**	**CAU**<br>**(Median, IQR)**	***p*-Value**
PANAS-C-PA<br>(*n* = 49, 55)	56 (48, 62)	52 (44, 60)	0.1126
MAAS-C<br>(*n* = 51, 58)	43 (29, 59)	45 (33.75, 55.25)	0.5518
SCS-C<br>(*n* = 51, 60)	38 (34, 43)	39 (36, 45)	0.3230
SDQ-T composite total difficulties score<br>(*n* = 62, 62)	6.500 (1.75, 13.00)	6.500 (2.000, 12.00)	0.9056
Internalizing subscale	9.500 (7, 10)	9.500 (6, 10)	0.7935
Emotion subscale	3.000 (0, 6.250)	1.000 (0, 4)	0.2172
Peer problems subscale	1.000 (0, 4)	0.000 (0, 3)	0.2337
Externalizing subscale	0.5000 (0, 3)	0.000 (0, 2)	0.5067
Conduct subscale	3.000 (0, 8)	4.000 (1, 9)	0.6805
Hyperactivity subscale	0.000 (0, 3)	0.000 (0, 2)	0.2381

Median scores were calculated for the baseline survey of HappiGenius and CAU classrooms. The Mann-Whitney test was used for all comparisons. The baseline survey scores were statistically similar between HappiGenius and class as usual. CAU, class as usual; PANAS-C-PA, Positive and Negative Affect Scale for Children—Positive Affect only; MAAS-C, Mindful Attentive Awareness Scale for Children; SCS-C, Self-Compassion Scale for Children; SDQ-T, Strengths and Difficulties Questionnaire—Teacher completed.

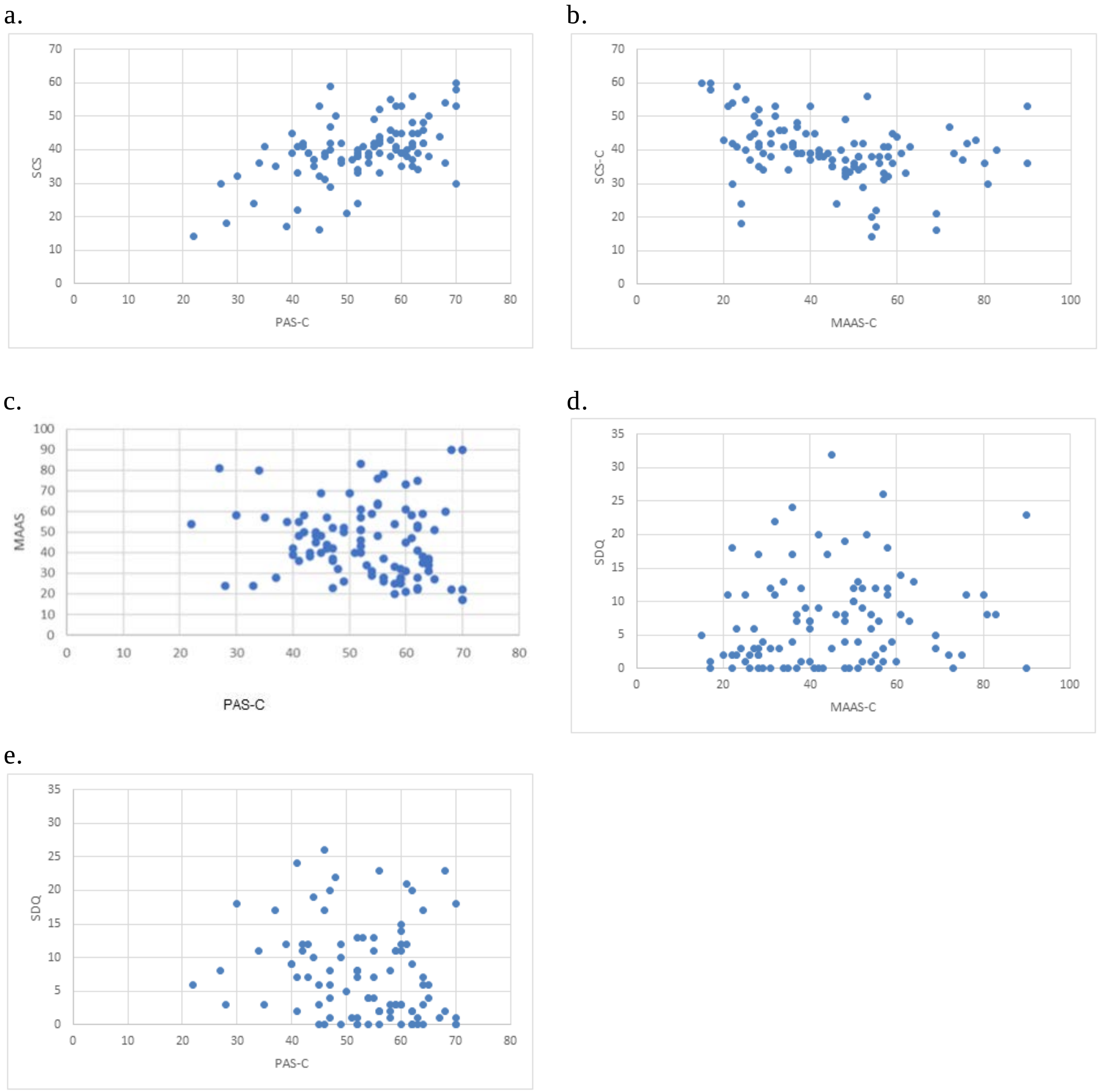

Figure S1 • Correlation analyses. A graphical representation of the significant correlations identified between (a) SCS-C and PAS-C; (b) SCS-C and MAAS-C; (c) MAAS-C and PAS-C; (d) SDQ total difficulties score and MAAS-C; and (e) SDQ total difficulties score and PAS-C. PAS-C, Positive Affect Scale for Children (positive affect only); MAAS-C, Mindful Attentive Awareness Scale for Children; SCS-C, Self-Compassion Scale for Children; SDQ, Strengths and Difficulties Questionnaire (teacher completed).

References

1. Laurent J, Catanzaro SJ, Joiner Jr TE, Rudolph KD, Potter KI, Lambert S, et al. A measure of positive and negative affect for children: scale development and preliminary validation. Psychol Assess. 1999;11(3):326–38. doi: 10.1037/1040-3590.11.3.326

2. Sutton E, Schonert-Reichl KA, Wu AD, Lawlor MS. Evaluating the reliability and validity of the self-compassion scale short form adapted for children ages 8–12. Child Indic Res. 2018;11(4):1217–36. doi: 10.1007/s12187-017-9470-y

3. Lawlor MS. Mindfulness and social emotional learning (SEL): a conceptual framework. Handbook of mindfulness in education. mindfulness in behavioral health. New York, NY: Springer; 2016. p. 65–80. doi: 10.1007/978-1-4939-3506-2_5

4. Goodman R. The strengths and difficulties questionnaire: a research note. J Child Psychol Psychiatry Allied Discip. 1997;38(5):581–6. doi: 10.1111/j.1469-7610.1997.tb01545.x

5. van den Heuvel M, Jansen D, Stewart RE, Smits-Engelsman BCM, Reijneveld SA, Flapper BCT. How reliable and valid is the teacher version of the Strengths and Difficulties Questionnaire in primary school children? PLoS One. 2017; 12(4):e0176605. doi: 10.1371/journal.pone.0176605
